# Bibliographical Notices

**Published:** 1837-04-01

**Authors:** 


### Periscope;

OR,

### CIRCUMSPECTIVE REVIEW.

"Ore trahit quodcunque potest, atque addit acervo."

### Notices of some New Works.

A Practical Treatise on the Principal Diseases of the Lungs, &c. By G. H. Weatherhead, M.D.

Dr. Weatherhead's great object in this little work is to attract more attention to the *particular tissues* affected in pulmonary diseases, than is usually given in this country. The volume, though small, is divided into ten chapters: viz. 1st, on catarrhal affections—2d, on phthisis—3d, on dry catarrh—4th, on the cough of measles—5th, on gouty cough—6th, on asthma—7th, on pleuritis—8th, on pulmonitis—9th, on hooping-cough—10th, on laryngitis. In all of these chapters Dr. W. introduces some peculiar opinions, especially of a theoretical nature—but often of a practical tendency. These, of course, are dispersed through much that is already well known to the profession, as elementary knowledge. To the French pathologists, and to some English physicians, (Carswell for example,) Dr. Weatherhead is much indebted, and candidly acknowledges the obligations. A work of this kind is not calculated for analysis; but as a specimen, not at all selected as a favourable one, we shall give the fifth chapter—a very short one—for the benefit of our readers.

"Of Gout in the Lungs; or Gouty Cough.

There are two forms under which gout not unfrequently appears in the lungs; the one of an acute character, the other of a chronic. The first begins with the ordinary symptoms of bronchitis, a violent, dry, and noisy cough, and slight febrile symptoms. After a time, the gout takes up its ordinary position by attacking the foot, the seizure, for the most part, coming on in the night. From the moment the disease makes its appearance in the regular form, the cough sensibly abates, and soon ceases altogether. Patients whose fits of gout are apt to return about the latter autumnal and during the winter months, are most liable to be thus affected; and with some it is so usually the harbinger of their periodical attack, that, adopting the wise maxim, '*Venienti occurrite morbo,*' they treat the disease in the lungs the same as if it were in its regular place—the foot.

A circumstance characteristic of this kind of cough is, that, while the patient remains in a warm room, the cough is little troublesome, and often will cease altogether for hours; but the slightest change of temperature, such as going into the passage, or up-stairs to a bed-room, is certain to bring on a violent fit of coughing. This excessive sensitiveness of the lungs to atmospherical variations is not peculiar, it is true, to gouty cough; but it possesses this particularity about it, that the degree of sensitiveness, and that of the inflammatory action, are not co-ordinately proportionate.

The other kind of gouty cough I spoke of, or the chronic, is confined in its attacks to those old and infirm subjects whose constitutions have been broken up by long-repeated aggressions of the disease. This form of gout does not fly to the extremities, but remains fixed in the lungs, while the paroxysm lasts. It is of a much more dangerous character than the first-mentioned species; and, assuming, as it is eventually apt to do, the pituitous character, it kills the patient in the ordinary manner of this disease—that is, by the destructive reaction of its own consequences—by the gradually increasing debility augmenting the quantity of phlegm, while the copious secretion of the latter reciprocally augments the debility: the air-cells becoming filled with mucus, which, from weakness, the lungs cannot expectorate, excludes the air inspired from coming in contact with the blood; till, at length, the phlegm accumulates in such quantity as to produce slow suffocation, and the vital powers become extinguished in consequence of a reacting series of destructive causes and effects.

With respect to the treatment of the first form of the disease, nothing so effectually relieves the pulmonary affection as colchicum. A demulcent mixture (24), containing colchicum and ipecacuan wines, may be taken during the day; and a full dose of colchicum, combined with some carbonate and sulphate of magnesia, at bed-time. This practice I have repeatedly seen, not merely relieve the lungs from the gout, but sometimes remove the paroxysm entirely.

Now, though the plan of treatment to be adopted, in chronic gouty cough, be somewhat similar, it is infinitely seldom so successful. We are to avail ourselves of the aid of colchicum in this form of the disease, also; but, as the expectoration rather requires to be checked than promoted, we must dismiss the ipecacuan, and substitute one of the preparations of morphia, or hyoscyamus, in its stead. The disease is to be solicited to the extremities by warm foot-baths, mustard poultices, and the like; and it is to be gently expelled from the chest by the employment of external derivatives. The regimen, which, in the first kind of gouty cough, requires to be somewhat severe, is in this, on the contrary, to be rather generous and sustaining. The body is to be warmly clad at all seasons of the year; and all the emunctories of the system kept in due activity, by regulating them by appropriate means, as they happen to require it."

The reader will find some novel and interesting views of *inflammation* in this volume, which views have been still farther developed by the author in a paper read at the Medico-Chirurgical Society at one of the late meetings.

---

PRACTICAL OBSERVATIONS ON NERVOUS AND SYMPATHETIC PALPITATION OF THE HEART. By JOHN CALTHROP WILLIAMS, M.D.

The design of this work is fully set forth in the title page, in which it is stated to be, the establishment of a diagnosis between palpitation of the heart, unattended by any appreciable change of structure, and that which is the result of organic disease. The author calls our attention to the extreme importance of every physician thoroughly understanding each symptom, which can assist him in determining what the actual state of the heart may be, in all cases of palpitation; and upon this point he has dwelt with much force and propriety. In strongly urging the necessity of this knowledge, Dr. Williams is not contending with an imaginary deficiency, on the part of his medical brethren, in their powers of diagnosis; especially when the symptoms pointed out by auscultation, are but imperfectly understood; and sufficient care and judgment are not exercised in combining such symptoms with those discovered by other means. We agree with Dr. W. in the justice of the following forcible remarks of Dr. Baillie: "There are indeed few pheno-

---

(24) ℞. Vini Sem. Colch.

—— Ipecac. āā ʒj.

Mucil. Acac. ℥iiss.

Mist. Amygd. ℥v. M.

Capiat æger cochl. amp. duo quater in die.

mena which puzzle, perplex, and lead into error the inexperienced (and sometimes the experienced) practitioner so much as inordinate action of the heart. He sees, or thinks he sees, some terrible cause for this tumult in the central organ of the circulation, and frames his portentous diagnosis and prognosis accordingly. In the pride of his penetration he renders miserable for a time the friends,—and by his direful countenance damps the spirits of his patient. But ultimate recovery not seldom *disappoints* his fears, and the physician is *mortified* at his own success."

When Dr. Baillie wrote this, the stethoscope was not used: but, will any candid practitioner deny, that it is an accurate description of what is even now of daily occurrence; sometimes through ignorance of our art, and, sometimes, from the deficiency of the means of diagnosis, even by the most skilful? A question however arises, whether in such a case as that just detailed, where functional has been mistaken for organic disease of the heart, the patient's fears are always *disappointed*, or the physician *mortified by success*. May he not by his error, and consequent mis-application of remedies create, or, at least allow the creation of, the very disease, which previously existed only in his own imagination; and which, but for his error, might have been prevented? The author says:

"In *nervous or sympathetic* affections of the heart, that abnormal action denominated palpitation forms a very prominent, and often the only feature; and is frequently, by a careless observer, regarded as symptomatic of some serious organic or structural change being established, either in the coverings of the heart, its muscular texture, or in some of its valvular appurtenances. * * * But it may be asked—does not disordered function, though in itself of trivial import, become in the course of time positive disease? Will it not merge into, or insidiously produce positive disease of vital organs, especially of that important organ the heart?" This opinion is confirmed "by the experience of Avenbrugger, Corvisart, Esquirol, Hope, Forbes, and other pathological writers." p. 6-7.

The transition of mere functional, into organic disease of the heart being established, we have an additional motive for learning every symptom, however slight, which may assist us in our diagnosis of these, often difficult, cases. If functional disease be mistaken for organic, in nine cases out of ten the treatment must be such as to keep up the deranged action of the heart; and a disease, at first slight and easily remediable, is converted into one which is incurable, and almost necessarily, though after the lapse of an uncertain period, fatal.

A conviction of the truth and importance of the foregoing observations, has induced us to give a diligent perusal to Dr. Williams's book; and we now recommend it with confidence to our readers, as a useful guide, in this most difficult part of medical practice, to those who are not well skilled in the diagnosis of diseases of the heart; and a work from which the most experienced and skilful may derive useful information.

The first part of the work is devoted to a description of the stethoscope, directions for using it, and an account of the natural and morbid phenomena arising from the heart's action. On all these points the author does not profess to give any new information; but he states what is known, clearly and concisely. As being closely and essentially connected with palpitation, when not arising from organic disease, "sympathy" has received considerable attention from Dr. W.; and this part of his subject he has treated with much judgment, avoiding what is vague and uncertain, in the various theoretical explanations of sympathetic connexions between different parts of the body; and confining himself to matters of fact; he has related shortly, but clearly, all that is actually known on this subject, which can be rendered serviceable in practice. We regret that we cannot give extracts from this part of the work, as the whole section is so connected, that any paragraph, taken by itself, would not do justice to the author.

In the second part of the work we find described, with great accuracy and clearness, the constitution and temperament of those who are the most liable to be affected with nervous palpitations, as well as a careful enumeration of all those accidental circumstances which, independently of any constitutional tendency, may give rise to the same derangement. The author then proceeds to detail, with much minuteness, all those symptoms which occur in palpitation, as it arises in persons of full habit and robust constitution, but in whom there is some local affection with which the heart sympathises; in persons of a directly opposite temperament; in persons who have generally good health, but are of a highly nervous and excitable temperament; in chlorotic females; in females who have lately ceased to menstruate; in those who have some irritation of the spinal cord; and in those who have some organic disease of the heart. In each of these cases the whole train of symptoms is described; and the points in which they differ from each other, according as they arise from one or other of the above causes, are detailed with all the care which the importance of the subject demands.

A few extracts will shew how the author has treated this part of his subject. After stating that there are cases of *active nervous palpitation* of the heart, connected with a plethoric condition of the system, which, in their early stages, closely resemble hypertrophy, he says: " A stout, ruddy-faced, plethoric country girl, consulted me in consequence of the extreme inconvenience she experienced from the over action of her heart. It beat full and strong against her ribs, and with sufficient force sensibly to raise my head at each stroke, when the stethoscope was applied over the præcordial region, though this is not usually the case. To this constant state of inordinate action, were added occasional attacks of palpitation, so tumultuous as scarcely to admit of analysis." In this case the uterus appeared to have been in fault, as menstruation had not occurred for several periods, and all the symptoms disappeared on the proper administration of antiphlogistic remedies. The author proceeds thus: " Although at first sight a train of symptoms such as just enumerated, might suggest the possibility of hypertrophy, yet on examining the phenomena collectively, the two disorders can scarcely be confounded, and might be distinguished even if the hypertrophy were complicated with palpitations. In the organic disease, the impulse of the heart is hard, circumscribed, and heavy; it raises the head of the observer at each stroke. The sound, instead of being clear, is obscure, and, as it were, muffled. A careful examination with the stethoscope will generally detect some irregularity in the contractions of the various chambers of the heart, either in respect to order or force. Percussion over the præcordial region, in advanced stages, produces a dull sound, which is not met with when the dimensions of the organ are natural. In hypertrophy, when the palpitations are, pro tempore, suspended, the beat of the heart still remains more hard and circumscribed than it ought to be, and indeed the movements of the heart are not always of a nature to merit the name of palpitation; they are not necessarily more rapid than in health—the patient does not always perceive them himself—and when he does, although they may in reality be much more forcible than natural, he seldom complains of the sensation being a source of distress, unless when the capacity of the chest is diminished by pressure, position, or otherwise. He attaches no undue importance to his symptoms, he evinces no unnecessary alarm respecting them, and even in the advanced stages of the complaint, seems insensible to his danger." P. 69—71.

In speaking of what is called "*passive palpitation*," he says, " in this variety of disease, the pulsations of the heart are increased in velocity, but not in strength; though apparently they seem so, they are not in fact. In many instances they are so slight as scarcely to be perceptible to the observer; yet they are always felt sensibly by the patient himself. When the morbid sensibility of the system is very great, an impulse, even much below natural, may be so annoying as to create considerable distress." P. 73. "In nervous palpitations, some well marked constitutional symptoms characteristic of the temperament is generally observed; in females, hysteria, with a morbid susceptibility to ordinary impressions; in males, hypochondriasis, or melancholy tendency; in both, great fear on the part of the patient as to immediate danger of life. Indeed there are not many diseases which excite in the mind of the patient so much alarm." P. 84.

The author gives six cases illustrative of palpitation without organic disease, and remediable, produced by the following causes: unexpected melancholy intelligence; tight lacing and spare diet; misfortunes in business; dyspepsia; the irritation of tapeworms; and checked uterine secretion. He then calls our attention to a species of palpitation occurring at the change of life in females; and remarks, that although this is generally connected, at that period, with a plethoric state of the system, it may arise from a condition directly the reverse. He also refers to a cause of palpitation independent of disease of the heart, which too often escapes notice—irritation of the spinal cord. Of this he gives the following case. "A young lady of delicate organization and susceptible system, had been ill more than two years, and the general, as well as pectoral symptoms, caused a belief she was consumptive. A careful examination by percussion and the stethoscope, induced a different opinion in my mind, and on drawing the fingers down the spinal column, a small lateral curvature was detected with considerable pain on pressure; *the pressure on the spine brought on pain in the chest, palpitations and cough.* Leeches, frictions, and fomentations to the spine, relieved the tumultuous movements of the heart more than any other remedies." At the end of a year, this patient, having removed to the country, and continued these remedies, was materially improved in health.—P. 104.

The observations upon the altered sounds of the heart in nervous palpitations, and the directions for distinguishing these from sounds almost similar, occurring in organic disease, are quite equal to the other parts of the work.

The author concludes by a few sensible and practical remarks upon the treatment of the various functional derangements he has described in the first part of the book.

---

THE HUNTERIAN ORATION, DELIVERED IN THE THEATRE OF THE ROYAL COLLEGE OF SURGEONS IN LONDON, ON THE 14TH FEBRUARY, 1837. By Sir BENJAMIN C. BRODIE, Bart. F.R.S. Serjeant Surgeon to the King, and Surgeon to Saint George's Hospital. Octavo, stitched, pp. 38. London, 1837.

The Hunterian Oration excited this year more than the ordinary share of curiosity and of attention, from the circumstance of the period of its delivery being selected, for the opening of what we may almost term the New College of Surgeons. The old building has been extended, altered, and improved, and the present temple of surgery is worthy of the profession, if not of the science.

There is a striking contrast between the Colleges of Physicians and of Surgeons. The mansion of the former, dark, dingy, and deserted, is a symbol of the fortunes of its masters. Obstinately resisting innovation, declaring by successive votes their adhesion to the past, the Fellows of that College have to all appearance sealed their corporate doom. No doubt there are many amongst them, of enlarged views, and of liberal feelings, who have struggled in vain against a prejudiced majority, and in vain endeavoured to secure the fortunes of their order. But that majority has unfortunately been too strong, and conservative in name, has proved destructive in reality.

The College of Surgeons have shewn much more willingness to march with public opinion, and reforms in its polity have been made with wisdom and alacrity. It would be well, and no doubt it would also be consistent with the ultimate stability of the Institution, if some modifications could be introduced into the mode of electing the Council, and if the government of the College could be made more popular than it is at present.

It is impossible to refrain from remarking the results of the cry for Reform that has agitated the profession for the last few years. It has been a subject of complaint, that the officers of the Colleges were not elected by the commonalty, and that the profession at large had no voice in the selection of its dignitaries. This was the great ground of remonstrance—this was the wrong that was most bitterly complained of—this it was that Parliament was called upon to remedy. The Parliament did interfere—its committee laboriously collected evidence, and reported on it—the King's Government took up the matter—and what has been the consequence? A new faculty, formed on the principle of centralization, and under the thumb of the Minister—a Faculty more independent of the profession and of popular election, than the councils of the old colleges. Observe that we do not pronounce an opinion, we do not say that the system is good or bad, we merely state the fact. It is essentially a system of centralization, it is essentially *not* a popular one. The Secretary of State appoints the members of the Faculty—the profession have nothing to do with his election. Now a Secretary of State can know little or nothing of professional merit, and he must depend on others for the information he requires. An opportunity is afforded for private influence and for intrigue, which, while human nature continues what it is, will pretty certainly be seized.

But to revert to the College of Surgeons. Sir Benjamin informs us, that:

" The library at this time contains not less than 20,000 volumes, and includes the most valuable works in the various branches of medicine, and in general science. Besides being open in the early part of the day as formerly, it will be open on three evenings of the week; an arrangement, which will, as I conclude, be very convenient to those gentlemen who are much engaged in practice.

The walls of the former museum have been elevated, so as to admit of a second gallery; and an additional building having been erected for the purpose of receiving the pathological collection, there will now be an opportunity of displaying many things relating to natural history and comparative anatomy, which were not displayed before. Various osteological specimens which formerly reposed in boxes in the vaults of the College, have already emerged from their obscurity, forming an extensive series of skeletons, and the whole is rendered at the same time more interesting and more intelligible by the *catalogue raisonné* which is now advancing rapidly towards its completion.

The lectures illustrative of the museum, have hitherto been consigned to different members of the College, none of whom have found it convenient to retain the office of professor for more than three or four years; and who, being engaged in other laborious duties, were seldom able to do full justice to the task which they had undertaken. Those lectures will now be delivered by the junior conservator, Mr. Owen, whose zeal and talents are known to you all; and who, devoting himself entirely to anatomy and physiology, will prove, as I venture to predict, a more efficient professor than any of us, who have preceded him. Thus there will be established, by means of this great museum and the lectures, a school of what may be called 'the science of life,' such as has never existed in this metropolis before, and we may reasonably conclude that this will ultimately be productive of great practical results."

The theatre of the College has been re-built. It is much larger and more commodious than it was, but still it cannot be considered a *very* good one. This, we believe, the Council could not help. The building has its limits, and something was judiciously sacrificed for the museum and the library. It is only on particular occasions that the theatre will prove too small—on ordinary ones, it will probably be too large.

Our space is limited, and we have little room for extracts from the interesting Oration which lies in print before us. It was received with great applause by a crowded audience, and not the slightest source of the cordial reception of the orator, was the good feeling that he displayed throughout. We will snatch a few passages, containing anecdotes of the illustrious dead.

*Partnership of William Hunter and Cullen.*

" A century has just elapsed since a young man, established as a medical practitioner in the then small town of Hamilton, in Scotland, received into his house another young man, not many years junior to himself, as a pupil, that he might instruct him, as far as his limited means gave him the opportunity of doing so, in the elements of medicine and surgery. After the lapse of three years, an intimate friendship having become established between the master and the pupil, it was agreed that they should enter into a partnership to practise in Hamilton as surgeons and apothecaries. For this purpose, however, it was thought necessary that the younger of these individuals should visit the medical schools, which had then been only lately established, in Edinburgh and London, so as to complete his education. Accordingly, we find him, at the end of another year, studying Anatomy in London under Dr. Douglas, a celebrated anatomist of that day, and filling, at the same time, the office of preceptor to Dr. Douglas's children. Here new schemes of life were offered to his ambition; and the result was, that he never returned to Hamilton. Not long afterwards his friend followed his example, seeking a wider field for the exercise of his talents, first in Glasgow, afterwards in Edinburgh.

Of these young men the one, and the elder, was William Cullen; and the other was William Hunter: and such was the humble origin of two of the most remarkable men who ever engaged in the pursuit of the medical profession."

*William Hunter's Enthusiasm as an Anatomical Lecturer.*

" It is natural for a man to delight in that occupation, in which he is conscious that he excels: and accordingly we find, that the delivery of his Lectures on Anatomy was William Hunter's favourite pursuit. At first, he found it convenient to teach anatomy, as affording him the means of subsistence; but he continued to do so, when the more lucrative pursuits of private practice had given him wealth beyond the most sanguine expectations of his early life. From this time, as I have been informed on good authority, he was accustomed to say, ' I wish to make no profit of my Lectures: I am quite satisfied if they pay their own expenses;' which, of course, included those of the anatomical department of his museum. The performance of his duties as a lecturer was terminated only by his death; and I have been informed that, when his last moments had arrived, his mind still reverted to that which he regarded as the most worthy occupation of his life, and that he said, ' I wish now that I had but strength to bear being carried into my theatre, that I might tell my pupils how much comfort and happiness I feel.' "

*Private Habits of William Hunter.*

" If we inquire into the private life of William Hunter, we find that he was never married; that his domestic establishment was on a very limited scale, and that his habits were of the most frugal kind. But let us not suppose that this was in any degree the result of avarice. He had no taste for those luxuries, or for that of display, for which wealth is usually exchanged; or, at all events, he had another object in view, which he prized more highly than these: and, in whatever related to this, he was liberal even to extravagance. I need scarcely say that I refer to his museum. I have already had occasion to mention that part of it which related to anatomy. It was collected for the purpose of illustrating his Lectures, and its chief value consisted in the preparations illustrative of the changes of the gravid uterus in those relating to diseased structure, and in the catalogue."

"But many present remember, as I do, that the anatomical department occupied only the gallery of the building in which the museum was placed. A most valuable and extensive library, and a costly collection of medals and minerals, filled the lower part of it, and served to demonstrate that the collector of these treasures could well estimate the value of other branches of knowledge, as well as those in the pursuit of which he was himself engaged. There was a collection of paintings also by the first masters, but deposited in other apartments. It is said, that the expense of forming the museum did not amount to less than 100,000*l.*, the whole of which was derived from the savings of a laboriously earned professional income."

*Manuscript Notes of John Hunter.*

"Although between forty and fifty years have elapsed since the death of John Hunter, there is one part of his labours in anatomy and physiology which has only lately become known to the public. He left behind him some manuscript notes, intended to be introductory to the study of the several departments of his museum. These are now being published in the catalogue; and they exhibit, in a remarkable degree, those powers of observation, of tracing resemblances and analogies, and of generalization, which belong to genius, and lay the foundation of discoveries. It cannot be doubted that an accomplished writer would readily convert these notes into essays, not less interesting and instructive, and certainly containing more original matter, than the introductory chapters which we read with so much delight in Cuvier's lectures."

*John Hunter's Labours not duly known.*

"He loved knowledge for its own sake. As I have already mentioned, he went on collecting his observations respecting the vascular system and inflammation, and yet had never published them, when he died at the age of sixty-five years. When I was formerly giving lectures as Professor of this College, I found in a drawer of the museum what appeared to be some pieces of dried sticks. Mr. Clift said that he did not know what they meant, but he was sure that they meant something, and therefore that he had preserved them. When I examined them, I found that they were the result of some interesting experiments in vegetable physiology. It appeared, from one of them, that he had made the first and most important of the experiments made many years afterwards by Mr. Andrew Knight, proving the descent of the sap through the vessels of the bark. Yet these specimens had no ostensible place in the museum; and they would have been swept away as rubbish but for the care of Mr. Clift. He solved the difficult problem of the circulation of the crustaceous animals, which others since his time, and Cuvier among the rest, have endeavoured to solve in vain. These things could not be displayed in preparations: the drawings representing them, with the explanatory notes appended to them, lay concealed in a cabinet; and I doubt whether more than half a dozen persons were acquainted with their existence, until I exhibited them in my Lectures, delivered in this College, in the year 1821."

We recommend the Oration to our readers. It will beguile a weary hour, and they will find Sir Benjamin a pleasant Cicerone, while they stroll among the tombs of some of our most celebrated predecessors.

---

COMPENDIUM OF LITHOTRIPSY, OR AN ACCOUNT OF REMOVAL OF THE STONE FROM THE BLADDER WITHOUT INCISION; ADAPTED FOR GENERAL COMPREHENSION, WITH A SERIES OF STATISTICAL TABLES AND NUMEROUS WOOD-CUTS REPRESENTING THE MOST IMPORTANT INSTRUMENTS AND IMPROVEMENTS UP TO THE PRESENT TIME. By HENRY BELL-

NAYE, Esq. &c. &c. 8vo. pp. 215. Bailliere, London, 1837.

The object of this little work is a very laudable one—to diffuse amongst the Profession and the public an acquaintance with the process and the merits of lithotripsy.

It is difficult to reconcile two aims, which in some respects differ widely from each other—that of instructing the public and the Profession. A popular work must avoid details, and explain in a simple, yet forcible, and in some degree an exaggerated manner, the principles of what it professes to teach. But the Profession is necessarily acquainted with those principles, detects the exaggeration, and requires the details which would not be intelligible to the public.

We cannot reasonably expect M. Belinaye to conquer difficulties inherent in his subject, and from their nature almost insurmountable. He has not conquered them. If any thing has been established, it is the fact that lithotripsy *is* a painful operation. M. Belinaye, however, cannot be expected to proclaim this. He observes—

"Many patients do not express even the least sensation of pain during the percussion." 85.

"To show how readily the lithotrite acts upon, and removes a small stone, a case has fallen beneath our notice lately, where the patient after a minute's endurance of the instrument, felt so little sensation and its magic was so rapid, that his mind became possessed by an opinion, that the whole affair was a mere jugglery, of which he had been the dupe. The remembrance of his having been sounded by several surgeons previous to the operation—the sight of the detritus of stone that had passed from him—and his presence at another equally rapid operation on another individual, brought home to his mind conviction, and congratulation at having, without suffering, escaped the knife." 118.

Such passages as these, and we might select more, must be admitted by those who have seen much of the operation to give but an imperfect idea of what is actually suffered, in the majority of instances, by those who undergo the lithotriptic process.

A comparison is instituted between lithotomy and lithotripsy. We cannot be surprised if that comparison should be tinctured with an obvious leaning towards the latter process. It is an amiable feeling, and it is impossible to avoid respecting the inclination to overrate what promises the relief of human misery. But time, which determines most practical questions, is gradually and surely solving this. As usual, truth will probably be found to lie in the mean between the extremes of the two opposing parties. One thing is quite certain. Lithotripsy has secured for itself all cases of small stones in healthy adults. The great aim of the benefactor of his species must be, to impress upon the public the necessity of attending to the symptoms of calculus in its early stage, and of applying in the first instance for surgical advice. Lithotripsy has, in this point of view alone, conferred inestimable advantages upon society.

Not the least interesting part of the work, is the author's account of the successive improvements which have been effected in the lithotriptic instruments. They prove, what is indeed known, that no art springs at once into maturity and perfection. But it has been a subject of complaint, that M. Belinaye has rather devoted himself to the praise of Baron Heurteloup, than to a strictly impartial history of lithotripsy. We will not affect to deliver an opinion upon this point. To a person unacquainted with medicine, the impression would undoubtedly be given that Heurteloup was almost the only man who had effected any material improvements in the process. That may be so. But Civiale, Leroy, and many others may feel indisposed to confirm the partial award of friendship. That award has not been niggard. The following quotation will shew that Baron Heurteloup receives his due, and "the puny and snarling companions," and "mosquitoes," are treated with very little ceremony.

"As for ourselves, we have been moved both for the love of the art of lithotripsy itself, and by a sense of duty to

Baron Heurteloup, to uphold his great share in its invention. Contrary to what has been the case with many other inventors, particularly in this branch of art, his process has been open to the study of every medical man —his operations performed in private practice before his brethren—in hospitals, before the whole world. And as if this were not enough, all men of respectability who have sought him in his privacy, have had opportunities of investigating every minutia—of putting questions—making captious objections to draw out information, &c. &c.—as we ourselves have done.

In this, therefore, we have had our personal share of obligation: we should be most happy to think that this day, by our poor efforts, we had repaid it—by contributing something to the fame and triumph of one, who, open to all fair objections of scientific and honourable men, has held on the even tenor of his way, silent amidst incessant attacks of mouth and pen dictated by ignorance, envy, and malice. Not unlike in this to the manner in which we see in nature, creatures of a nobler race and growth, move amidst the punier and snarling companions with which they are obliged to herd.

The misfortune is that you cannot deal as easily with the malignancy of petty enemies, as you do with underhand detractors. You quietly despise the latter until time awards justice; but the former, like musquitoes, with their skin-deep wounds and eternal buzzing, distract your attention and delay your progress."

We must again observe that we do not pretend to enter on this controversy. Indeed our attention has been chiefly directed to it by a letter which we have received from Mr. Costello, and which we think it only justice to that gentleman to publish. With this we take leave, for the present, of the subject.

*To the Editor of the Medico-Chirurgical Review.*

Sir,—Allow me to call your attention to a work on lithotrity, just published, under the name of Mr. Belinaye, in which the author highly extols his countryman, M. Heurteloup, on the score of *his* inventions. With this gentleman's sentiments of admiration, or the mode he has taken to make them known, I have nothing to do, but I must object in the strongest manner to the exercise of a liberality which would deprive me of the merit of certain inventions of the lithotritic apparatus, which belong to me, and which he, (perhaps through simple ignorance of facts—for I will not impute improper motives), attributes to his friend.

M. Belinaye gives the drawing of several instruments which he says exhibit a regular "system of Baconic Induction." I must suppose, that the author here means that every successive modification of the instrument has led M. Heurteloup to further improvement, and thus that every new device in this course of instrument-making had carried him, one step nearer to perfection; and as we learn that in these attempts, he must have made "a room-full of instruments," the plain inference is, that the links in this long concatenation, must be exceedingly numerous indeed. The point of perfection, or goal of all these labours, is at length attained, and within a very recent period—a few months perhaps, the art of lithotrity has been enriched, by the invention of two instruments—the one, a lithotrite without teeth—the other a spoon, or spoon-beaked instrument.

As M. Belinaye undertook the responsibility of historian, he ought to be aware, that these two instruments were invented, and presented by me to the profession in 1832; the whole series, from the imperfect model which I obtained from Weiss, to the perfect instrument which is now in general use both in France and England, amounting to four only. These instruments were exhibited at the Westminster Society—to the Grand Institute of France immediately after—and a drawing of the toothless instrument was published in the Lancet.

Much doubt has been expressed by surgeons of eminence as to the possibility of terminating an operation of lithotrity, and cases have been referred to, in which fragments have been left in the bladder. In a letter, signed, "The Shade of Le Dran," in the Lancet, a case of this kind forms the groundwork of a charge against lithotrity, to which I felt compelled to reply. I did there promise, by anticipation, to defend lithotrity from the charge which is applicable to defective instruments, and by no means to the process ; and I now redeem that pledge.

I have always objected to instruments having large lateral incisive teeth—always maintained that instruments should be so constructed, that if, through want of skill or tact on the part of the surgeon, the attempt to perform the operation should prove fruitless, the patient should be left in no worse condition than he was before the attempt had been made—in a word, if the instrument could accomplish no good, it should do no harm. It was for these reasons that I invented my double slide break-stone, without teeth.

With an instrument armed with cutting teeth, it is not possible to terminate safely the treatment of a case. The fragments, when few and small, lie flat on the surface of the bladder; and when an instrument with teeth is employed to gather them up, there is the greatest risk of wounding and pinching the viscus. It was to obviate this danger, that I constructed the spoon-beaked instrument, which I have used constantly in clearing the bladder of fragments since 1833. In one case of lithotrity performed on Mr. Job, watchmaker, residing at Camden Town, it was indeed my chief resource, as the patient was affected with paralysis of the bladder and lower extremities. The fragments thus obtained, and now in possession of Mr. Job, fill a four-ounce vial.

M. Belinaye will, I am sure, excuse me for thus vindicating my claim to those instruments. They are now universally adopted to the exclusion of "roomfuls" of other modifications.—Indeed Dr. Labbatt of Paris, in his visit to this country two years ago, had the candour to assure me, "that without my instrument the practisers of lithotrity must have fallen back on Dr. Civiale's instruments." I am however happy to perceive, that my confrere, M. Heurteloup is a convert to opinions, and instruments, which I have not ceased to recommend for the last five years. I honestly believe, that much of the disgrace that has fallen on lithotrity is attributable to the defective construction and principles of the instruments that have hitherto been so extensively and injudiciously employed, but now that all parties are agreed as to the superior value of my instruments, I would fain believe, with M. Belinaye, that the same number of mishaps will not occur in the course of a century.

In conclusion, I have only to express a hope, if M. Belinaye should write again on this subject, that he will see the propriety of respecting the principle of "*suum cuique*."

I remain, Sir, your's, &c.

W. B. COSTELLO.

---

### Recent Publications on Anatomy.

Several articles have this quarter so far exceeded the limits we had calculated on, that others must be postponed until the following number of this Journal. That is our apology for the mere mention we can now make of some recent works. They will receive a fuller notice in our next.

The Works upon anatomy that have appeared since our last have been :—

I. The *Second Part of Mr. Swan's Illustrations of the Nervous System.* The plates are equal (no slight praise) to their predecessors. They represent —the dorsal aspect of the brain and spinal cord of the cod—the sympathetic nerve of the skate—the dorsal aspect of its brain and spinal cord—the dorsal aspect of its nervous system—the brain and spinal cord of the turtle. Of the letter-press we shall hereafter speak at large. In the mean time we strongly recommend the work.

II. *Sketch of the Comparative Anatomy*

*of the Nervous System, with Remarks on its Development in the Human Embryo, &c. &c. By J. Anderson, M.E.S. &c.* This is a succinct account of the nervous system, illustrated with drawings, and elucidated by diagrams. It too demands a fuller notice at our hands, and will receive it. We recommend it to our readers.

III. *The Anatomy of the Human Body, illustrated by a Series of Engravings. With Letter-press. By P.D. Handyside, M.D.* These are chiefly selected from Fyfe's Plates. It may be doubted whether they are equal to the sets now in process of publication by Bourgery, Quain, &c.

IV. *The late Fasciculi of Quain's Plates.* The subscribers have every reason to be satisfied with these. Their execution is improved, not degenerating. Let there be enough of them.

V. *On the Connexion of the Anterior Columns of the Spinal Cord with the Cerebellum. By Samuel Solly, Esq.* This has been noticed a few pages further on. Mr. Solly deserves great credit.

NOUVEAU SYSTEME DE PHYSIOLOGIE VEGETALE ET DE BOTANIQUE, &c. Par F. V. RASPAIL.

This will be amply reviewed in our next Number. It is a very valuable work.

---

### NEW PHARMACEUTICAL WORKS.

1. *The New Pharmacopœia* has made its appearance and has been duly worried by the chemists. It is a difficult matter to please these gentlemen. Save us from being concerned with them.

2. *Translation of the New Pharmacopœia by Mr. Phillips.* This appears to us to be a very good one. Mr. Phillips has been violently assaulted, but he has evinced much gentlemanly feeling and good humour.

3. *Translation of the New Pharmacopœia by Dr. Spillan.* There is room both for Mr. Phillips and Dr. Spillan. The latter gentleman, like all the *pharmaciens*, has come in for a little "raillery" in the Tony Lumpkin style. Yet his translation is a good one.

---

### PRINCIPAL ORIGINAL PAPERS PUBLISHED IN THE WEEKLY AND MONTHLY BRITISH JOURNALS, BETWEEN THE 1st OF JANUARY, AND THE 20th OF MARCH, 1837.

We have no great love for catalogues. Knowledge we conceive to be essentially a substantial thing—practical knowledge certainly is so. A pedant is content to learn what others have done, *because* they have done it—what is said or written *because* it is written or said. To him it matters little whether the thing is good or bad. He does not seek or love it for its merit, but takes it as a pioneer takes a trull, because she is a trull.

A quarterly journal may be made a bibliographical record. Three hundred pages may contain an account of three hundred books and papers. But what an account? a copy of their titles—an ex cathedrâ opinion on their merits! Can such a plan give the substance of the works of value? The question answers itself—it is preposterous to suppose that either journal or plan can do any thing of the sort.

The mass of the profession we may reasonably suppose to be actuated with the wish to possess at a cheap price, what is really valuable in the current medical literature. The body of practitioners are not mere book collectors or idle virtuosi. They do not keep a ledger, in which to post that John-a-nokes has written a long paper on the adaptation of rhubarb and magnesia to children's bowels—or that Dr. Hopingarter has published a most elaborate thesis, on the best method of cutting one's toe-nails. The body of the profession have something else to do, and something better, than to waste their leisure on this abuse of learning, or on carefully collecting these cobwebs of the brain. They are

Hard-handed men, who labour here in Athens—

and their time is too precious, let us add that their sense is too sound, to permit them to cram their literary craws with such indigestible pedantic pastry.

We are plain matter-of-fact folks, and looking at things in that point of view which regards their utility, we can see little benefit in filling our pages with a multitude of mere notices, which can*not* be much more than abstracts of tables of contents. It is useless to give merely a sketch of a good book—and it is worse than useless to give a sketch of every bad one.

Our own opinion, we speak as utilitarians, is this:—*that* what is really desired in a periodical, is the publication in that cheap form which periodical literature only can afford, of what is real and sterling information—*that*, for this purpose, an analysis of a few good books and good papers, is preferable to a million notices of as many volumes—*that* to diffuse information amongst the mass of the profession, the necessities of that mass and its tastes should be consulted—*that* the object of a philosophical critic should be, to diminish those necessities, and to elevate that taste—*that* mere bibliography and transcendentalism are adapted to do neither—but *that* the inculcation of a purer love of facts, of a reasonable dread of theory, of a close and accurate observation, and of a spirit of exact generalization, is calculated to serve the best interests of medicine and of medical practitioners.

Such is our creed—such is the system we have acted on and shall act on. Let others think as they may and do as they think, we have yet seen no reason to depart from this line of action. We have often professed ourselves, we do so still, utilitarians—utilitarians not in a sordid but a philosophic sense—utilitarians, not merely deeming—

> ——the value of a thing,

> To be whatever it will bring; but looking on it to ascertain whether it is or is not calculated to increase that stock of substantial knowledge, which is so satisfactory in itself and in its consequences.

Consistently with this declaration of our sentiments, we put in a subordinate rank and place, our bibliographical notices. But though subordinate, we shall render them complete, and our readers will find at the termination of each quarter, a list of the works that have been published, and the original papers that have appeared during its course. One word upon the latter. We must exercise on their enumeration, some degree of despotism. In the weekly Journals there are many communications, which can hardly be called original papers. They are explanations, or nameless sorts of epistles, from that nameless class of persons, who appear under the *soubriquets* of verax, justitia, and so forth—designations frequently adopted on the lucus à non lucendo principle, because the writers display an utter contempt both for justice and truth. With this explanation, we proceed statistically.

### I. Anatomy.

*Medical Gazette.*

On the height of the diaphragm; by Dr. Edwin Harrison.—Jan. 7.

Account of an anomalous distribution of the branches of the aorta; by Francis Hird, Esq.—Feb. 4.

### 2. Physiology.

*Medical Gazette.*

Observations on the second sound of the heart; by T. Watson, M.D.—Jan. 7.

On the causes of the pectoral resonance in percussion, with experiments; by T. Clendinning, M.D.—Jan. 7.

Is taste a special sensation? being an answer to Mr. Noble's late remarks; by Dr. B. Alcock.—Jan. 7.

On the prevalence of calculous disorders among seamen; by J. Johnson, M.D.—Jan. 7.

On the organic globule—a physiological memoir; by Dr. T. G. Hake.—Jan. 14.

On the functions of Meckel's ganglion and the glosso-pharyngeal nerve; by M. W. Hilles, Esq.—Jan. 14.

Third report of medical cases treated at St. George's Hospital; by R. Macleod, M.D.—Feb. 4.

Remarks on influenza; by Henry Bullock, Esq.—Feb. 4.

Influenza, as it has prevailed in the House of Correction; by H. Wakefield, Esq,—Feb. 4.

Discovery of ciliary motions in the cavities of the brain; by Parkinge.—March 11.

*Lancet.*

Case of precocity in the mammæ of an infant; by W. Howitt, Esq.—Jan. 7.

On the formation of new substances, or the transmutation of old ones, by vegetable, animal, and chemical processes; by Mr. James Inglis.—March 18.

3. Materia Medica, and Practice of Medicine.

*Dublin Journal.*

On the hydriodate of potash as an emmenagogue, in a letter from Dr. Pinching.—Jan.

Researches on the symptoms and diagnosis of aneurismal and other tumors in the cavity of the thorax; by G. Greene, M.D.—Jan.

Contributions to practical medicine; by Dr. Piez.—Jan.

Observations on cerebral and spinal apoplexy, paralysis, and convulsions of new-born infants; by Evory Kennedy, M.D.—Jan.

Case of hydrophobia; by Richard Long, M.D.—Jan.

*Medical Gazette.*

On some prominent novelties in the new edition of the London Pharmacopœia; by T. Everitt, Esq.—Jan. 7.

On the efficacy of chlorine in scarlatina; by R. T. Taynton, Esq.—Jan. 28.

Reply to Mr. Everitt's remarks on the New Pharmacopœia; by R. Philips, Esq.—Feb. 4.

On certain errors of Laennec and others respecting the regions of the chest; by Edwin Harrison, M.D.—Feb. 18.

Notice of the influenza of January and February, 1837; by John Clendinning, M.D.—Feb. 18.

Notes on the influenza; by R. Macleod, M.D.—Feb. 18.

Remarks on the nature and treatment of rheumatism; by T. Hope, M.D. Feb. 25.

On the character of the prevailing influenza; by W. W. Morgan, Esq. Feb. 25.

On abscess of the liver; by R. H. Allnatt, Esq.—Feb. 25.

Influenza at the St. Marylebone Infirmary; by T. Clendinning, M.D.—Feb. 25.

Efficacy of the strychnos nux vomica in affections of the digestive organs; by T. Mellor, Esq.—March 4.

A few remarks on molloscum, with two cases of molloscum non-contagiosum; by W. Dick, Esq. March 4.

Erysipelas, its nature and appropriate treatment; by H. Bullock, Esq.—March 4.

*Lancet.*

Remarks on Mr. Everitt's criticism of the New London Pharmacopœia; by R. Philips, Esq.—Jan. 14.

Case of carcinome sanglante, with remarks on the nature and origin of the non-analogous productions. By H. A. Roberts, Esq.—Jan. 28.

Case of pericarditis, ending fatally, with remarks on the effects of bloodletting in disease of the heart; by John Fosbroke, M.D.—Feb. 4.

New Pharmacopœia. Further remarks by Mr. Philips.—Feb. 4.

Case of scalding of the glottis cured with calomel; by Geo. Fayrer, Esq. —Feb. 11.

Case of hydrophobia; by T. G. Davey, Esq.—Feb. 18.

Remarks on the influenza; by W. Wansborough, Esq.—Feb. 25.

New views on hectic fever; by C. W. Edwards, Esq.—Feb. 18.

On the external application of opium in bronchitis and croup; by W. Bow, M.D.—March 18.

4. Surgery.

*Dublin Journal.*

A case of circumscribed false aneurism of the brachial artery, caused by puncture in bloodletting, and cured by operation; communicated by Thomas Wall, Esq.—Jan.

*Medical Gazette.*

Cases in operative surgery, with remarks; by F. C. Skey, Esq.—Jan. 7.

Cases of dislocation of the head of the femur; by T. Wormald, Esq.—Jan. 28.

Account of a new method of operating for hydrocele; by Benjamin Travers, Esq.—February 11.

Sequel of a case of extra-uterine pregnancy, in which a full-grown fœtus was removed by an operation fourteen months after conception, with remarks; by F. Hutchinson, Esq.—February 11.

New method of treating hydrocele—in reply to Mr. Travers; by D. Lewis, Esq.—February 18.

Treatment of hydrocele by puncture and absorption; by Robert Keate, Esq.—February 18.

On the treatment of hydrocele by iodine; by Charles Caswall, Esq.—March 4.

*Lancet.*

Successful treatment of hydrocele by acupuncturation; by D. Lewis, Esq.—Jan. 14.

Case of "hydrocephalus externus," treated by puncture of the head; by J. Jeffrey, Esq.—January 21

Case of severe injury to the thigh; by J. P. Oates, Esq.—January 21

Operations for the removal of abdominal tumors; by R. C. King, Esq.—January 21

Case of malignant disease of the testis in a child; by D. W. Brown, Esq.—January 28

Case of traumatic tetanus, successfully treated by oil of turpentine—Jan. 28

New mode of operating for strangulated hernia, to obviate the danger of wounding the hernial sac; by M. W. Hilles, Esq.—February 11

Case of empyema, the effusion finding vent externally; by T. W. Macnee, Esq.—February 11

Failure of acupuncturation in hydrocele—February 18

Case of hydrocele cured by acupuncture; by Dr. Hacket—February 25

Case of strangulated hernia, relieved without wounding the peritoneum; by H. Meyers, Esq.—February 25

Case of strangulated hernia, treated according to Mr. Key's practice of dividing the stricture externally to the hernial sac; by Mr. W. Howitt—March 4

On the treatment of simple exostosis by repeated blistering; by Mr. W. F. Denham—March 18

### 5. Pathology.

*Dublin Journal.*

Letter from Dr. Perry on typhus fever.—January
Pathological contributions, No. 1; by Alexander John Hannay.—January
Some remarks on epidemic typhus fever, which prevailed in the parish of Donoughmore, in March, April, and May, 1836; by T. Henderson Babington—January

*Medical Gazette.*

On the contagiousness of typhus fever, and on the elements of medicine; by Dr. R. Williams—January 7
Case of an extensive encysted hepatic abscess with hydatids; by R. H. Allnatt—February 4
A remarkable case of gangrene of the right lower extremity, supposed to originate in a diseased state of the heart; by William Clark, Esq.—February 11
A combination of pathological states concurring in the same individual—Hydatids and their origin; by Dr. T. Ogier Ward—March 4
Case of hyperencephalus monstrosity, conjoined with other monstrous formations; by Walter Dick, Esq.—March 11
Case of obstructed ureter, with ulceration of the kidney and abscess; by R. Allan, Esq.

*Lancet.*

Account of the influenza prevailing among horses, with remarks on its treatment; by Charles Clark, Esq.—January 17
Observations on idiopathic chronic arachnitis; the diseases of the serous membranes; and the diagnosis, termination, and treatment of idiopathic chronic arachnitis, with cases; by Mr. M. W. Hilles—March 4

*Medical and Surgical Journal.*

A succinct treatise on medical reform for Ireland; by W. Stoker, M.D.—January 7

Dr. Sander's contribution to the pathology of the heart—February 4

### 6. MIDWIFERY.

*Dublin Journal.*

Cases of placenta presentation; by W. Jamieson, M.D.—January

*Medical Gazette.*

Midwifery practice; placental presentation and expulsion; by E. Augustus Corey, Esq.—January 28

On placental presentation; by J. Collier, M.D.—March 4

### 7 MEDICAL JURISPRUDENCE.

*Medical Gazette.*

Reports of the medico-legal examination of the trunk of a human body lately found in the Edgeware-road; by G. F. Girdwood, Esq.—Jan. 7

Report of an examination of the head of a female found under mysterious circumstances, with remarks; by G. F. Girdwood, Esq.—January 14

Medico-legal report on the lower extremities of a human body, found under mysterious circumstances, with remarks; by G. F. Girdwood, Esq.—February 11

### 8. MISCELLANEOUS.

*Medical Gazette.*

The medical witnesses' act; its injurious tendency; by F. H. Ramsbotham, M.D.—January 7

On self-supporting dispensaries; in reply to Mr. Smith; by W. Eales, Esq.—January 7

Medical statistics—On the identity of the vie probable and the vie moyenne, as deduced from the English population returns—January 28

Royal Institution—Dr. Faraday on a new general law of matter—Jan. 28

Observations on the mode and the amount of clinical and other medical instruction at Paris; by Edwin Harrison, M.D.—February 4

Anomalous condition of assistant-surgeons in the navy, with an address to Sir W. Burnett—February 4

Observations on the comparative mortality of the rich and of the poor; by John Rickman, Esq.—February 18

*Lancet.*

Remarks impeaching the capacity of Mr. Finlaison as the author of the annuity tables; by T. K. Edmonds, Esq. B.A.—January 14

Note from Mr. Baxter on the position of the patient in the treatment of vesico-vaginal fistula—January 7

Defensive remarks on Mr. Liston's operations for the removal of the superior maxillary bone—February 4

*Medical and Surgical Journal.*

Letter from Mr. Phelan respecting the progress of reform, and of the medical commissioners—January 21

Sketches of the state of medical knowledge in Persia; by H. F. Clarke, Esq.—February 4

### 9. LECTURES.

Course of lectures on medical jurisprudence; by Professor A. T. Thomson, now being delivered at the University of London—*Lancet*

Course of lectures on the physiology of the nervous system; by M. Majendie, in 1836—*Lancet*

Clinical lecture on the pustular group of venereal eruptions; by Dr. Wallace—*Lancet*

Lectures on forensic medicine, delivered at the Aldersgate School of Medicine; by W. Cummin, M.D.—*Med. Gaz.*

Clinical lectures delivered at the Meath Hospital during 1836-7; by Professor Graves—*Medical Gazette*

Notes of lectures on physiology; by Dr. Fletcher—*Lon. Med. & Sur. Jour.*

Course of lectures on materia medica and therapeutics; by Dr. Sigmond—*Lancet*

Lectures on materia medica, or pharmacology and general therapeutics; by Jon. Pereira, Esq.—*Med. Gaz.*

